# Multifunctional Biomaterial Strategies to Regulate Inflammation and Promote Kidney Repair

**DOI:** 10.34133/bmr.0312

**Published:** 2026-01-27

**Authors:** Jeong Min Park, Jun Yong Kim, Boram Kim, Eun Hye Lee, Seung Yeon Lee, Sun Hong Lee, Duck Hyun Song, Won-Kyu Rhim, Jeoung Eun Lee, Tae-Keun Ahn, Bum Soo Kim, Dong Ryul Lee, Dong Keun Han

**Affiliations:** ^1^ Department of Biomedical Science, CHA University, Seongnam-si, Gyeonggi-do 13488, Korea.; ^2^ Department of Ophthalmology, CHA Bundang Medical Center, CHA University, Seongnam-si, Gyeonggi-do 13496, Korea.; ^3^ Joint Institute for Regenerative Medicine, Kyungpook National University, Daegu 41944, Korea.; ^4^ ORANDBIO Co., Ltd., Uiwang-si, Gyeonggi-do 16108, Korea.; ^5^ Bundang Medical Center, CHA Advanced Research Institute, CHA University, Sungnam-si, Gyeonggi-do 13488, Korea.; ^6^ Department of Orthopedic Surgery, CHA Bundang Medical Center, CHA University, Seongnam-si, Gyeonggi-do 13496, Korea.; ^7^ Department of Urology, School of Medicine, Kyungpook National University, Daegu 41944, Korea.

## Abstract

Chronic kidney disease (CKD) involves inflammation, fibrosis, and impaired regeneration. We developed a biofunctional hybrid scaffold (PMEAR/MM/uEV) combining a porous poly(lactic-co-glycolic acid)-porcine extracellular matrix, ricinoleic acid-modified magnesium hydroxide, metanephric mesenchyme-like cells, and ureteric bud-derived extracellular vesicles, with resveratrol and adapalene to confer antioxidant and pro-regenerative properties. The scaffold exhibited uniform porosity, pH-buffering, and reactive oxygen species-scavenging activity. In vitro, it accelerated epithelial wound closure, reduced oxidative stress, and shifted cytokine profiles toward an anti-inflammatory state by increasing interleukin-4 while decreasing tumor necrosis factor-alpha, interleukin-6, and interleukin-8. In a 5/6 nephrectomy mouse model, PMEAR/MM/uEV reduced collagen deposition, improved blood urea nitrogen and creatinine, and up-regulated podocyte markers synaptopodin, nephrin, and podocin, as well as the renal developmental marker Pax2. mRNA sequencing revealed activation of angiogenesis, extracellular matrix remodeling, oxidative defense, and immune modulation, with Kyoto Encyclopedia of Genes and Genomes enrichment in tumor necrosis factor and interleukin-17 signaling and nuclear factor kappa B-associated pathways. These findings establish PMEAR/MM/uEV as an effective, multimodal platform for kidney regeneration.

## Introduction

Chronic kidney disease (CKD) is a progressive condition marked by chronic inflammation, fibrotic remodeling, and impaired tissue regeneration—factors that current therapeutic options such as dialysis or kidney transplantation fail to address beyond temporary functional support [1]. The limited regenerative capacity of renal tissue, especially within nephron units, and the persistence of a pro-inflammatory microenvironment often result in irreversible damage [2]. Conventional treatments such as dialysis and kidney transplantation provide temporary functional restoration but do not address the underlying pathophysiological mechanisms or promote true tissue regeneration [3,4]. Consequently, there is a critical need for regenerative strategies that offer not only mechanical support but also active modulation of the biological milieu to enable functional tissue repair [5].

In recent years, biodegradable porous scaffolds have emerged as promising platforms for tissue engineering due to their ability to mimic the native extracellular matrix (ECM), enabling cell adhesion, migration, and spatial organization [6–8]. However, many synthetic scaffolds lack intrinsic bioactivity and do not adequately resolve oxidative stress or inflammatory signaling in injured kidneys, limiting their therapeutic applicability in CKD [9]. To address these shortcomings, hybrid scaffolds incorporating bioactive molecules, progenitor cells, and immunomodulatory agents are being explored to enhance regenerative capacity [10,11]. In this study, we developed a biofunctional hybrid scaffold (PMEAR/MM/uEV) designed to meet the multifactorial demands of renal tissue regeneration by integrating structural, biochemical, and immunological functions. The scaffold consists of a porous, biodegradable poly(lactic-co-glycolic acid) (PLGA) matrix functionalized with porcine-derived decellularized ECM, ricinoleic acid-modified magnesium hydroxide (MH-RA), metanephric mesenchyme-like (MM) cells differentiated from embryonically derived intermediate mesoderm, and extracellular vesicles derived from ureteric bud cells (uEVs). Each component contributes synergistically to therapeutic outcomes.

The decellularized porcine ECM provides essential structural proteins and growth factors that facilitate cellular adhesion, nephron lineage-specific differentiation, and vascularization [12,13]. MH-RA functions as a localized pH buffer, mitigating the acidic microenvironment associated with tissue injury and PLGA degradation, while magnesium ions and ricinoleic acid further contribute antioxidant and anti-inflammatory effects [14,15].

Recently, extracellular vesicles (EVs) have gained considerable attention in regenerative medicine as versatile therapeutic tools capable of modulating cellular responses and tissue repair [16–19]. A growing body of studies has demonstrated their potential in various organ systems, including the kidney, liver, heart, and nervous system [20–22]. EVs are nano-sized particles known to reflect the molecular characteristics of their parent cells, carrying bioactive molecules that influence intercellular communication and the local microenvironment [23–25]. The incorporated uEVs are nano-scale vesicles rich in regulatory RNAs and proteins that promote immune modulation, angiogenesis, and anti-fibrotic signaling [26,27]. Localized delivery via the scaffold improves the bioavailability and tissue targeting of uEVs, overcoming limitations of systemic EV administration [28–31]. In parallel, MM cells serve as nephron progenitor-like elements with regenerative potential under spatially defined conditions, contributing to tissue remodeling [32,33].

**Fig. 1.**
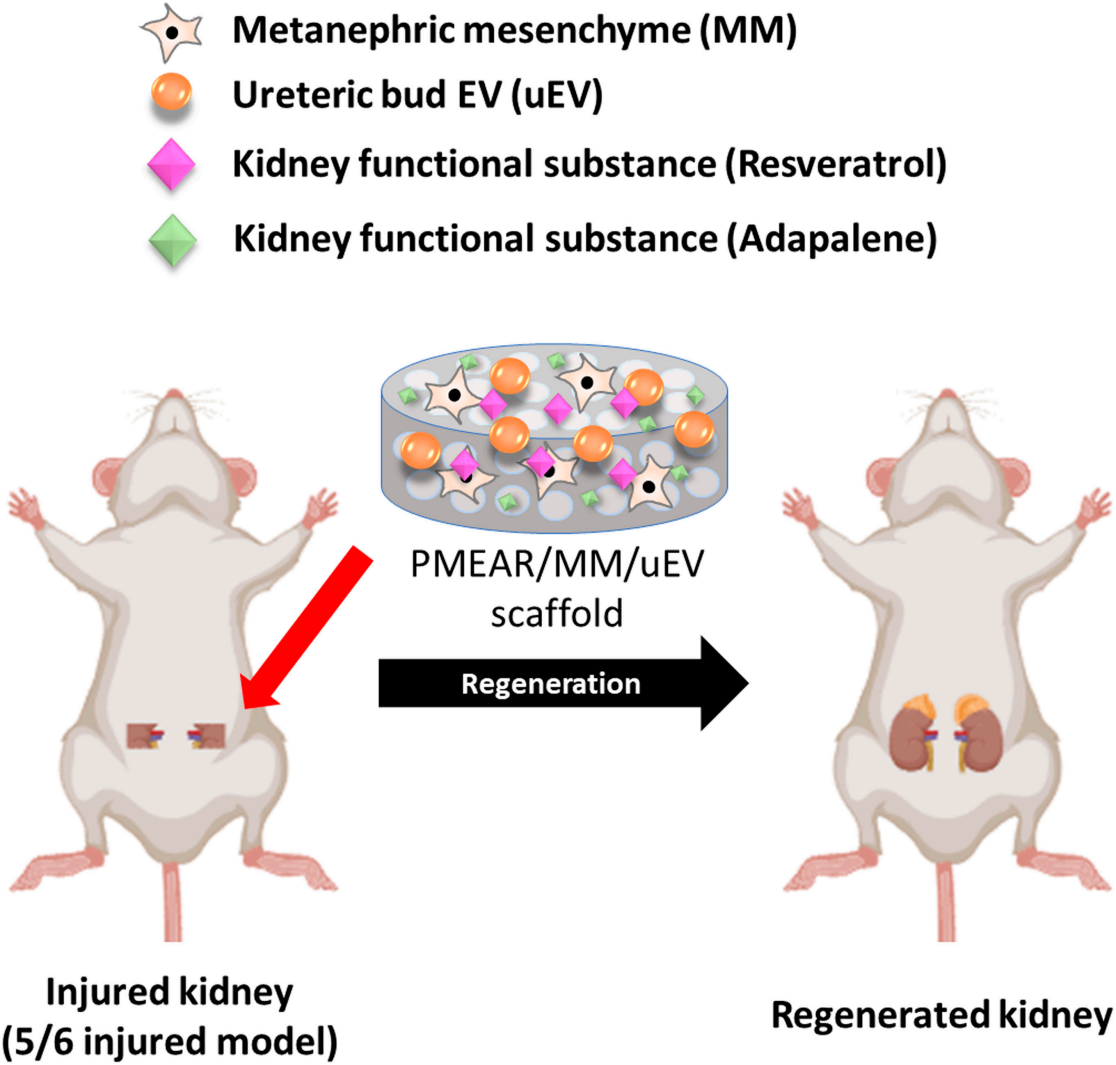
Schematic overview of the renal tissue regeneration strategy. The figure illustrates the overall experimental design of this study. A functionalized scaffold, referred to as PMEAR/MM/uEV, was constructed by incorporating multiple bioactive components poly(lactic-co-glycolic acid) (PLGA, P), magnesium hydroxide (MH, M), porcine-derived extracellular matrix (ECM, E), adapalene (A), resveratrol (R), metanephric mesenchyme (MM), and engineered ureteric bud-derived extracellular vesicles (uEVs). This composite scaffold was locally applied to a 5/6 nephrectomy mouse model to enhance renal regeneration.

We hypothesized that this multifunctional scaffold would reduce inflammation, attenuate fibrosis, and promote structural and functional kidney regeneration. To test this, we performed physicochemical characterization, in vitro cellular and molecular assays, and in vivo implantation in a 5/6 nephrectomy mouse model [34] (Fig. 1). Our findings demonstrate that the scaffold enhances wound healing, modulates inflammatory responses, and preserves podocyte integrity and glomerular architecture across both early and chronic stages of kidney injury. These results underscore the translational potential of hybrid scaffolds for regenerative therapy in CKD.

## Materials and Methods

### Materials

PLGA (LA/GA ratio 50:50, MW 40,000) was sourced from Evonik Industries (Essen, Germany). Magnesium hydroxide [Mg(OH)₂; MH-RA], retinoic acid (RA), adapalene, and resveratrol were obtained from Sigma-Aldrich (St. Louis, MO, USA), while ricinoleic acid was purchased from TCI (Tokyo, Japan). Primary antibodies against CD63 and Apo-A1 were acquired from Santa Cruz Biotechnology (Santa Cruz, CA, USA). Secondary antibodies, including anti-rabbit immunoglobulin G (IgG) and anti-mouse IgG, were obtained from Cell Signaling Technology (Danvers, MA, USA).

### Cell culture

Ureteric buds, generously provided by Dr. Dong-Youn Hwang, were cultured in CellCor CD MSC medium (Xcell Therapeutics, Seoul, Korea) supplemented with 1% antibiotic–antimycotic solution (A/A; Gibco, Grand Island, NY, USA) to produce ureteric bud-derived extracellular vesicles (uEVs). Human renal proximal tubular epithelial cells (HK2), obtained from the Korean Cell Line Bank (Seoul, Korea), were maintained in RPMI 1640 medium (Gibco) supplemented with 10% fetal bovine serum (Hyclone Laboratories, Logan, UT, USA) and 1% A/A.

### Isolation and characterization of EVs

EV isolation and characterization were conducted as previously optimized. For EV isolation, conditioned medium was prepared by collecting cell culture supernatants daily over a 5-day period. The supernatants were centrifuged at 1,300 rpm for 3 min at room temperature, followed by filtration through a 0.22-μm vacuum filter/storage bottle system to remove particles larger than EVs. EVs were then purified using a tangential flow filtration (TFF) system equipped with a 500-kDa molecular weight cutoff filter. The resulting EVs were resuspended in phosphate-buffered saline (PBS) and stored at −80 °C. Particle size and concentration were determined via nanoparticle tracking analysis (NTA) using the MONO ZetaView instrument with a 488-nm scatter mode (PMX-120, Particle Metrix, Meerbusch, Germany). EV morphology was examined by transmission electron microscopy (TEM; H-7600, 80 kV; Hitachi, Tokyo, Japan) following negative staining.

### Western blot analysis

Cells were lysed in radioimmunoprecipitation assay buffer supplemented with a protease inhibitor. Equal amounts of EVs (1 × 10^9^ particles) were resolved on 12% sodium dodecyl sulfate–polyacrylamide gels and transferred onto nitrocellulose membranes using transfer buffer. Membranes were blocked with 5% skim milk for 1 h at room temperature, then incubated overnight at 4 °C with anti-CD63 and anti-Apo-A1 primary antibodies (1:200 dilution). Following washes with Tris-buffered saline containing 0.05% Tween 20, membranes were incubated with horseradish peroxidase-conjugated anti-rabbit IgG or anti-mouse IgG secondary antibodies (1:2,000) for 1 h at room temperature. Protein signals were detected using enhanced chemiluminescence (GE Healthcare, Milwaukee, WI, USA) and imaged with the ChemiDoc XRS+ system equipped with Image Lab software (Bio-Rad, Hercules, CA, USA).

### Fabrication and characterization of scaffolds

Multifunctional polymer scaffolds composed of PLGA (P), magnesium hydroxide (MH-RA; M), extracellular matrix derived from human adipose tissue (ECM; E, Hans Biomed Inc.), adapalene (A), and resveratrol (R) were fabricated using the ice particle leaching method. Briefly, PLGA (0.25 g), MH-RA (15 wt%), ECM (20 wt%), A (50 μM), and R (25 μg) were dissolved and homogenized in dichloromethane (1.92 ml). Following freeze-drying to remove volatile organic solvents, 1 × 10^9^ uEVs were loaded onto hydrated scaffolds and stabilized for 24 h. Scaffold morphology was examined via field emission scanning electron microscopy (Sigma, Carl Zeiss, Jena, Germany) at an accelerating voltage of 5 kV, after gold sputter-coating for 30 s. The inorganic content was quantified by thermogravimetric analysis (TGA; PerkinElmer, Waltham, MA, USA) at a heating rate of 10 °C/min from 30 to 800 °C. Surface chemical bonds were analyzed by attenuated total reflectance–Fourier transform infrared spectroscopy (ATR-FTIR; Sigma, Carl Zeiss, Oberkochen, Germany) within the 650 to 4,000 cm^−1^ range at a scan speed of 0.2 cm/s. Scaffold degradation was evaluated by immersing individual scaffolds in 1 ml of PBS and incubating at 37 °C in a shaker (150 rpm). pH variations were monitored over 28 days using a pH meter (Mettler Toledo, Columbus, OH, USA). Antioxidant capacity was assessed via the 2,2-diphenyl-1-picrylhydrazyl (DPPH) assay, wherein 100 μl of each microsphere–hydrogel sample was mixed with 400 μl of DPPH working solution (250 mM) and incubated at room temperature in the dark for 1 to 7 days. Absorbance at 515 nm was recorded using a precision microplate reader.

### The mRNA sequencing of EVs and bioinformatics analysis

Small RNA libraries were prepared using the NEBNext Multiplex Small RNA Library Prep Kit (Macrogen, Seoul, Korea) according to the manufacturer’s protocol. Briefly, total RNA samples were ligated to adaptors, followed by cDNA synthesis using reverse transcriptase with adaptor-specific primers. Libraries were amplified via polymerase chain reaction (PCR) and purified using polyacrylamide gel electrophoresis and the QIAquick PCR Purification Kit (QIAGEN, Hilden, Germany). Library yield and size distribution were evaluated using the Agilent 2100 Bioanalyzer with the High-Sensitivity DNA Assay (Agilent Technologies, CA, USA). High-throughput sequencing was performed on the Illumina NextSeq 550 platform, generating single-end 75-bp reads. Functional prediction was carried out through Gene Ontology (GO) and Kyoto Encyclopedia of Genes and Genomes (KEGG) pathway analyses using the DAVID Bioinformatics Resources (https://davidbioinformatics.nih.gov/, accessed on 2025 August 10).

### Wound healing assay

HK2 cells were seeded in 6-well plates at a density of 1.5 × 10^5^ cells per well. After 16 h to allow cell adhesion, a straight scratch was created across the cell monolayer using a sterile 1-ml pipette tip. The scratched areas were gently rinsed twice with PBS (Welgene, Gyeongsan-si, Korea) to remove detached cells. Cells were then incubated at 37 °C for 16 h with PMEA, PMEAR, PMEAR/MM, or PMEAR/MM/uEV scaffolds. Following incubation, wound closure was visualized using an optical microscope, and the extent of wound healing was quantified with ImageJ software (National Institutes of Health, Bethesda, MD, USA).

### Real-time quantitative PCR of in vitro samples

Total RNA was extracted from cells using the AccuPrep Universal RNA Extraction Kit (Bioneer, Daejeon, Korea) following the manufacturer’s protocol. cDNA synthesis was carried out using the PrimeScript RT reagent kit (Takara, Shiga, Japan). Primer sequences for real-time quantitative PCR (RT-qPCR) were designed as previously reported. Amplification was performed with SYBR Green PCR reagents (Applied Biosystems, Foster City, CA, USA) on a QuantStudio 3 system (Applied Biosystems). Gene expression levels were quantified using the 2^−ΔΔCt^ method, with 18S rRNA serving as the internal control. All reactions were conducted in triplicate in 3 independent experiments.

### Designs for in vivo experiments

All animal procedures were approved by the Institutional Animal Ethics Committee of Kyungpook National University College of Medicine. Six-week-old female ICR mice were obtained from Jung Ang Lab Animal, Inc. (Seoul, Korea) and randomly assigned to experimental groups. A 5/6 nephrectomy model was established by surgically resecting 2- to 3-mm segments from both the apical and inferior poles of each kidney. At 2 and 8 weeks following scaffold implantation into the right kidney, mice were euthanized, and kidney tissues were harvested for subsequent analyses. Anesthesia was induced with 3% to 4% isoflurane and maintained at 1% to 3%.

### RT-qPCR of in vivo samples

Total RNA was extracted from kidney tissues using the Maxwell RSC simplyRNA Cell Kit with the Maxwell 16 instrument (Promega, Madison, WI, USA). One microgram of RNA was reverse-transcribed into cDNA using the GoScript Reverse Transcription Mix (Promega) following the manufacturer’s instructions. RT-qPCR was carried out on a StepOnePlus Real-Time PCR System (Applied Biosystems) using LUNA NEB SYBR Green Master Mix (NEB, MA, USA). Primer sequences were designed as previously reported.

### Histological and functional analyses

Kidney tissues were fixed in 10% formalin and embedded in paraffin. Sections of 4 μm thickness were mounted on coated glass slides. Hematoxylin and eosin (H&E), periodic acid–Schiff, and Masson’s trichrome (MT) staining were performed to evaluate general histology, glomerulosclerosis, and fibrosis, respectively. Stained slides were examined using light microscopy. For immunohistochemistry, sections were deparaffinized in xylene, rehydrated through graded ethanol, and subjected to antigen retrieval in citrate buffer. After blocking with 5% bovine serum albumin, primary antibodies (1:100) were applied for 18 h at 4 °C. Fluorescein isothiocyanate (FITC)-conjugated secondary antibodies were then applied for 2 h at room temperature. Slides were mounted with 4′,6-diamidino-2-phenylindole mounting medium (Vector Laboratories, Burlingame, CA, USA) and examined under fluorescence microscopy.

### Statistical analysis

Data were analyzed using GraphPad Prism version 9 (GraphPad Software, San Diego, CA, USA). Results are expressed as the mean ± SD. Unpaired *t* tests or one-way analysis of variance, followed by Tukey’s multiple comparison post-test, were used to analyze group differences. For all experiments, statistical significance was defined as *P < 0.05, **P < 0.01, ***P < 0.001, and ****P < 0.0001 for 3 biological replicates.

## Results and Discussion

### Fabrication and characterization of biofunctional hybrid scaffolds incorporating antioxidant and regenerative agents

To construct a biofunctional scaffold capable of promoting renal tissue regeneration while mitigating oxidative stress, resveratrol and adapalene were coloaded into a PLGA-based matrix. Resveratrol, a well-known activator of SIRT1, has been reported to enhance intracellular reactive oxygen species (ROS) scavenging and attenuate oxidative damage [35,36]. Adapalene, previously identified in our study as a renal differentiation enhancer, was included to potentiate regenerative outcomes.

To counteract the acidic degradation by-products of PLGA (P), magnesium hydroxide [Mg(OH)₂] particles surface-modified with ricinoleic acid (MH-RA; M) were incorporated as buffering agents. In addition, porcine-derived ECM (E) was used to mimic the native kidney microenvironment. The final scaffold, PMEAR, comprised PLGA (P), MH-RA (M), ECM (E), adapalene (A), and resveratrol (R). The final composite scaffold, referred to as PMEAR, was fabricated using a freeze-leaching method with ice particulates to create a highly porous structure.

**Fig. 2.**
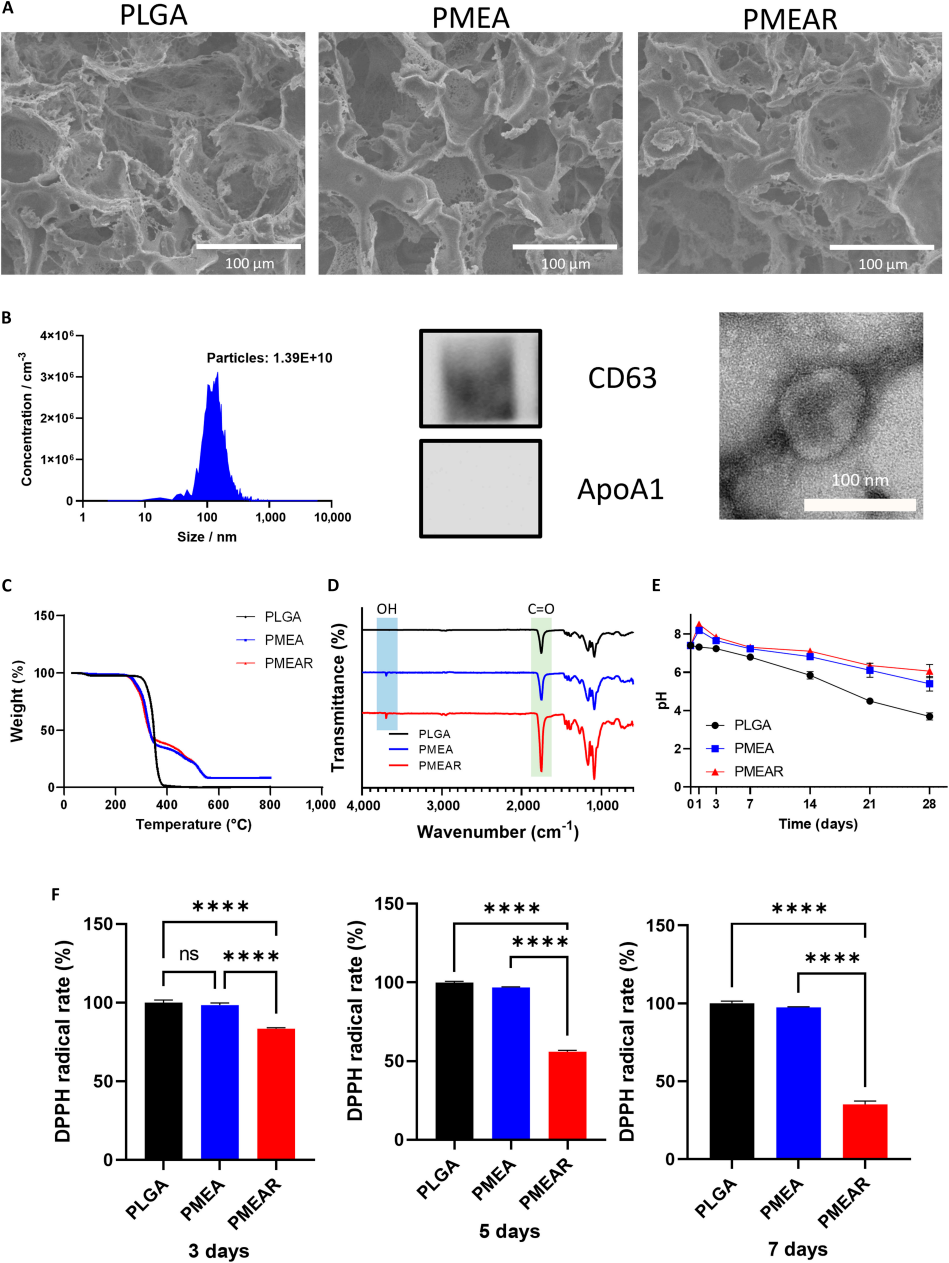
Characterization of the functionalized porous scaffold and incorporated extracellular vesicles (EVs). (A) NTA of uEVs showing size distribution and concentration (left), Western blot analysis confirming the expression of EV-associated markers CD63 and ApoA1 (middle), and transmission electron microscopy (TEM) images revealing the typical morphology and size of uEVs (right). Scale bars = 100 nm. (B) SEM images of PLGA, PMEA, and PMEAR scaffolds. Scale bar = 100 μm. (C) TGA thermograms. (D) ATR-FTIR spectra of PLGA, PMEA, and PMEAR scaffolds. (E) pH value changes during in vitro scaffold degradation. (F) DPPH radical scavenging activity of PLGA, PMEA, and PMEAR scaffolds.

To further enhance the regenerative microenvironment, EVs were isolated from embryonically derived ureteric bud cells (uEVs), known for their nephrogenic capacity. Ureteric bud cells were expanded under optimized 2-dimensional culture conditions with chemically defined media (CDM), and EVs were isolated via a TFF system, a method reported to improve vesicle yield and activity [26,37]. The concentration and size distribution of the isolated EVs were quantified using the MONO ZetaView system. To verify the identity of the isolated EVs, Western blot analysis was performed in accordance with the MISEV 2023 guidelines [38]. The EV-specific transmembrane marker CD63 was clearly detected, while the non-EV marker apolipoprotein A1 (Apo-A1) was absent, indicating successful EV isolation. TEM further revealed that the uEVs exhibited characteristic spherical and cup-shaped morphologies (Fig. 2A).

SEM analysis of the fabricated scaffolds confirmed uniformly distributed, interconnected pores across all compositions (Fig. 2B), indicating successful pore formation via the ice particle-leaching technique. Such architecture is favorable for cell infiltration and EV entrapment.

TGA was used to determine scaffold composition and thermal stability. Compared to PLGA-only controls, scaffolds containing adapalene and resveratrol displayed slower degradation rates (Fig. 2C), likely due to interactions between the incorporated molecules and the PLGA matrix, contributing to improved structural integrity.

Fourier-transform infrared (FTIR) spectroscopy further confirmed successful incorporation of MH-RA into the scaffolds. A prominent -OH stretching band observed between 3,700 and 3,500 cm^−1^ validated the presence of hydroxyl groups from RA-modified Mg(OH)₂ (Fig. 2D).

To assess buffering capacity, pH changes during scaffold degradation were monitored. While PLGA-only scaffolds exhibited progressive acidification over time, PMEAR scaffolds maintained a near-neutral pH (Fig. 2E), supporting the neutralizing function of MH-RA against acidic PLGA degradation products.

The antioxidant functionality of incorporated resveratrol was further verified by measuring intracellular ROS levels in H₂O₂-treated cells. Cells cultured on resveratrol-containing scaffolds exhibited a time-dependent reduction in ROS levels (Fig. 2F), consistent with previous studies demonstrating ROS-scavenging effects of resveratrol through SIRT1 activation [39]. This suggests that the antioxidant component may protect encapsulated or neighboring cells from oxidative stress during tissue repair.

Collectively, these results demonstrate the successful integration of key biofunctional elements including nephrogenic EVs, antioxidant and pro-regenerative agents, ECM, and buffering compounds into a structurally and chemically optimized hybrid scaffold. The PMEAR scaffold holds promise as a multifunctional platform for renal tissue regeneration under oxidative stress conditions.

### Expectation for the functionality of scaffold

mRNA sequencing was performed to elucidate the transcriptional mechanisms underlying the enhanced regenerative effects of PMEAR/MM/uEV scaffolds. Genes were considered differentially expressed when the absolute log base 2 fold change exceeded 1—meaning that their expression levels differed by more than 2-fold while meeting an adjusted *P* value < 0.05.

**Fig. 3.**
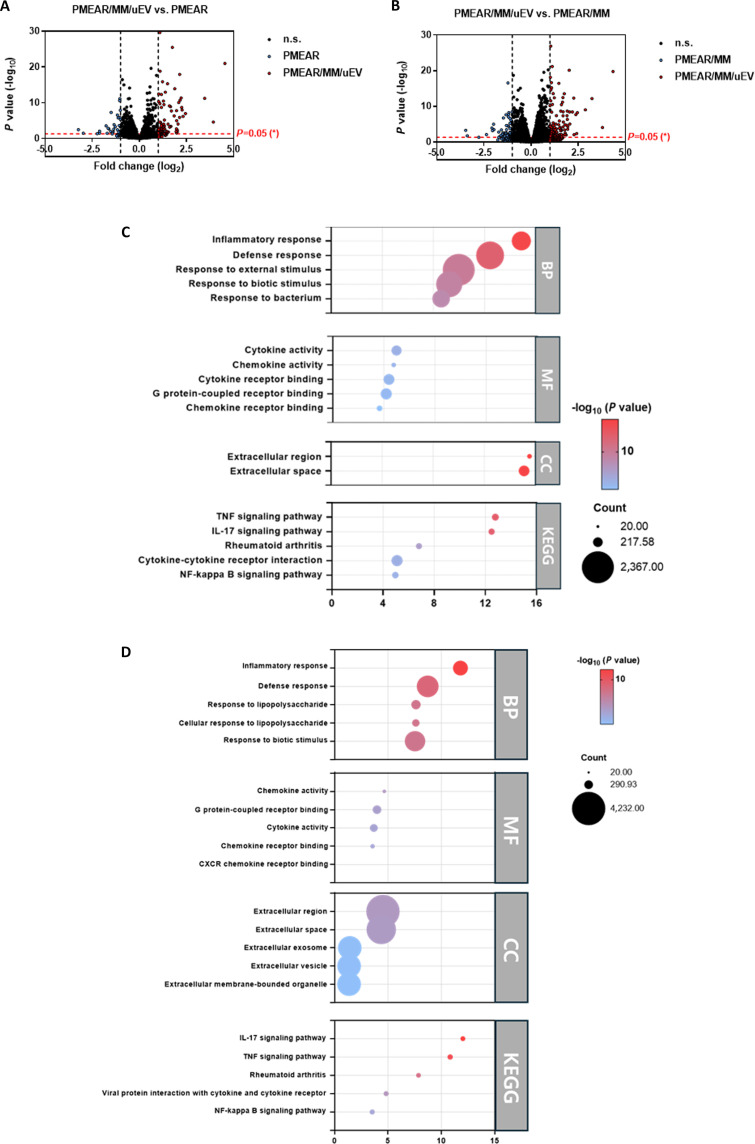
Transcriptomic profiling and functional enrichment analysis of PMEAR/MM/uEV scaffolds. (A) Volcano plot analysis (PMEAR/MM/uEV vs. PMEAR). (B) Volcano plot analysis (PMEAR/MM/uEV vs. PMEAR/MM). (C) Functional enrichment of DEGs: PMEAR/MM/uEV vs. PMEAR. (D) Functional enrichment of DEGs: PMEAR/MM/uEV vs. PMEAR/MM.

In the comparison between PMEAR/MM/uEV and PMEAR (Fig. 3A), a substantial number of differentially expressed genes (DEGs) were identified. Genes up-regulated in PMEAR/MM/uEV included TIE1, PLAT, TNC, MMP1, MMP7, LAMB3, IL6, CXCL2, CXCL3, CXCL5, CCL2, CCL20, CSF1, NFKBIA, CD40, BIRC3, SOD2, G0S2, CSF2, PTX3, and POU2F2. These genes are strongly linked to angiogenesis and vascular remodeling (TIE1, PLAT, and TNC) [40], ECM remodeling and stabilization (MMP1, MMP7, and LAMB3) [41], immune modulation and anti-inflammatory signaling (IL6, CXCL2, CXCL3, CXCL5, CCL2, CCL20, CSF1, NFKBIA, CD40, and BIRC3) [42], oxidative stress defense (SOD2 and G0S2) [43], and tissue repair-associated growth factor signaling (CSF2, PTX3, and POU2F2) [44]. These patterns indicate that incorporating both MM cells and uEVs drives a transcriptomic program favoring vascular regeneration, ECM stabilization, immune regulation, and cytoprotection.

When PMEAR/MM/uEV was compared with PMEAR/MM (Fig. 3B), where the only difference was the presence of uEVs, a distinct gene signature emerged. Up-regulated genes in the uEV-containing group overlapped with those linked to angiogenesis (TIE1, PLAT, and TNC), epithelial cell differentiation (LAMB3 and MMP7), oxidative stress defense (SOD2 and G0S2), and cytokine-mediated repair signaling (IL6, CXCL2, CXCL3, CXCL5, CCL2, CSF1, and TNFAIP3). This reinforces that uEVs deliver bioactive factors capable of amplifying vascular regeneration, epithelial restoration, oxidative protection, and cytokine-driven repair, providing a unique pro-regenerative stimulus beyond that of MM cells alone.

GO Biological Process (BP) analysis (Fig. 3C) revealed enrichment in “inflammatory response”, “defense response”, “response to external stimulus”, and “response to bacterium”, suggesting active modulation of host immune defense and injury resolution. GO Molecular Function (MF) terms included “cytokine activity”, “chemokine activity”, “cytokine receptor binding”, “G protein-coupled receptor binding”, and “chemokine receptor binding”, indicating enhanced intercellular signaling and immune cell recruitment. GO Cellular Component (CC) terms such as “extracellular region” and “extracellular space” reflect the secretion and extracellular localization of many up-regulated factors. KEGG pathway enrichment identified “TNF signaling pathway”, “IL-17 signaling pathway”, “Rheumatoid arthritis”, “Cytokine–cytokine receptor interaction”, and “NF-κB signaling pathway”, highlighting regulation of inflammation and immune-mediated tissue repair.

GO BP terms (Fig. 3D) were dominated by “inflammatory response”, “defense response”, “response to lipopolysaccharide”, “cellular response to lipopolysaccharide”, and “response to biotic stimulus”, pointing to modulation of innate immunity and pathogen defense. GO MF terms included “chemokine activity”, “G protein-coupled receptor binding”, “cytokine activity”, “chemokine receptor binding”, and “CXCR chemokine receptor binding”, which are indicative of chemotactic signaling and immune cell recruitment. GO CC terms such as “extracellular region”, “extracellular space”, “extracellular exosome”, “extracellular vesicle”, and “extracellular membrane-bounded organelle” reflect the EV-mediated delivery of these bioactive factors. KEGG pathway enrichment revealed “IL-17 signaling pathway”, “TNF signaling pathway”, “Rheumatoid arthritis”, “Viral protein interaction with cytokine and cytokine receptor”, and “NF-κB signaling pathway”, suggesting that uEVs influence key inflammatory and immune regulatory networks relevant to tissue repair.

Together, the DEG profiles and GO/KEGG enrichment analyses demonstrate that PMEAR/MM/uEV scaffolds activate a coordinated set of molecular programs involving angiogenesis, ECM remodeling, immune modulation, oxidative stress defense, and cytokine-mediated repair. The additional transcriptional effects observed when uEVs are incorporated indicate that they potentiate immune regulation and vascular regeneration beyond the contribution of MM cells alone, providing a strong mechanistic basis for their superior performance in kidney tissue regeneration.

### Biofunctional scaffold promotes wound healing and modulates inflammatory responses

**Fig. 4.**
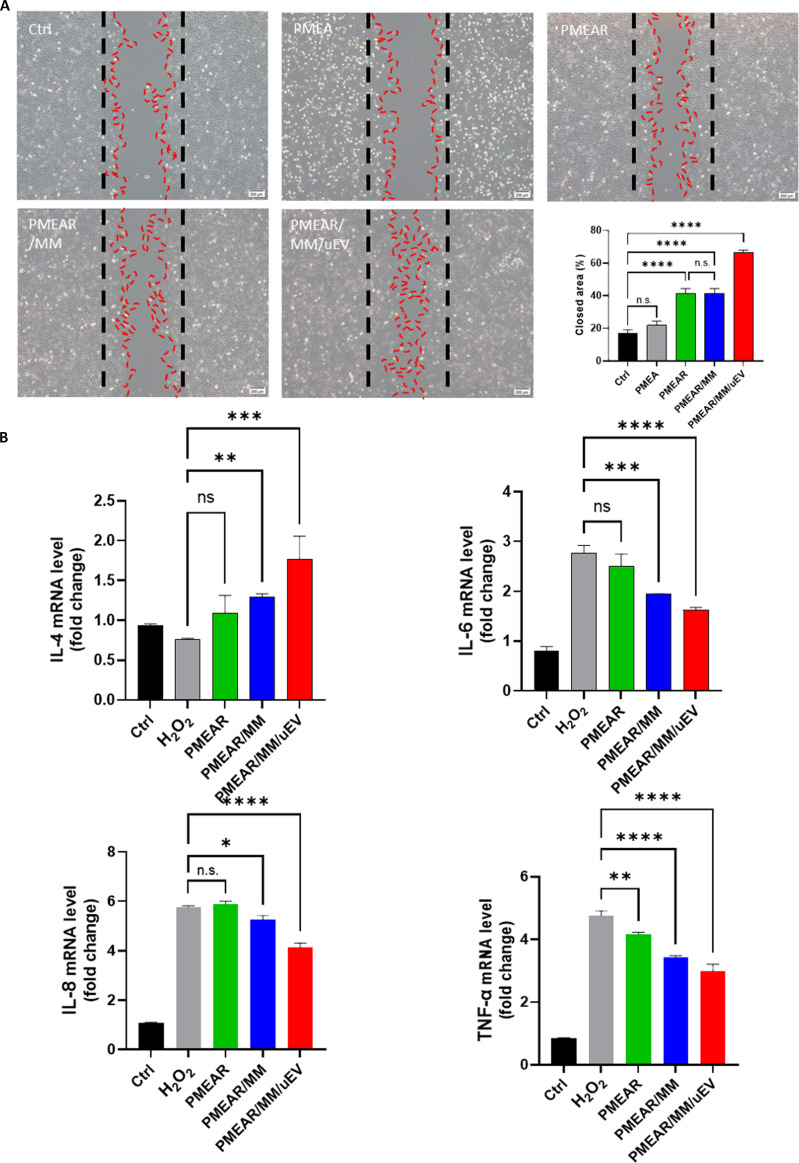
In vitro wound healing and inflammation effects of scaffolds. (A) Quantification of in vitro wound closure by renal epithelial cells following treatment with various scaffold formulations. Scale bars = 200 μm. (B) Relative expression of anti- and pro-inflammatory cytokines (IL-4, IL-6, IL-8, and TNF-α) under oxidative stress with scaffold treatment (*n* = 3).

To evaluate the regenerative and immunomodulatory performance of the biofunctional hybrid scaffold, we conducted both in vitro wound healing and inflammatory cytokine modulation assays. In a renal epithelial cell scratch model, the PMEAR/MM/uEV scaffold that integrates a porous PLGA-based matrix, porcine-derived ECM, RA-MH, resveratrol (R), adapalene (A), MM cells, and uEVs showed the highest wound closure rate compared to PMEAR and PMEAR/MM groups (Fig. 4A). Quantitative image analysis revealed that PMEAR/MM/uEV accelerated gap closure to more than twice that of untreated controls, suggesting that the combined presence of cellular (MM) and vesicular (uEV) components may contribute to enhanced epithelial migration and repair.

We next assessed the immunomodulatory capacity of scaffold under oxidative stress. HK-2 renal epithelial cells were pretreated with H_2_O_2_ to induce ROS generation and inflammatory activation, followed by culture on the different scaffold formulations. PMEAR/MM/uEV significantly increased interleukin-4 (IL-4) secretion while concurrently decreasing tumor necrosis factor-α (TNF-α), IL-6, and IL-8 expression compared to other groups (Fig. 4B). These changes reflect a pronounced shift toward an anti-inflammatory cytokine profile. Notably, scaffolds without MM or uEVs but containing resveratrol and adapalene also reduced TNF-α expression, consistent with literature reports that resveratrol suppresses NF-κB signaling and adapalene modulates retinoid receptor-mediated inflammation resolution [27]. This suggests that while bioactive molecules confer baseline anti-inflammatory effects, incorporation of MM cells and uEVs amplifies immune reprogramming toward a pro-regenerative phenotype.

Collectively, these data indicate that the PMEAR/MM/uEV scaffold not only enhances cell migration and wound repair but also actively modulates oxidative and inflammatory microenvironments, creating favorable conditions for kidney tissue regeneration.

### In vivo anti-fibrotic and anti-inflammatory effects of EV-loaded hybrid scaffolds in a 5/6 nephrectomy mouse model

A 5/6 nephrectomy mouse model was used to induce CKD and evaluate the therapeutic efficacy of scaffold [45]. After resection of the upper and lower poles of one kidney followed by a 2-week recovery period, the contralateral kidney underwent identical pole resection, and scaffolds (PMEAR, PMEAR/MM, or PMEAR/MM/uEV) were implanted at the injury site [11].

**Fig. 5.**
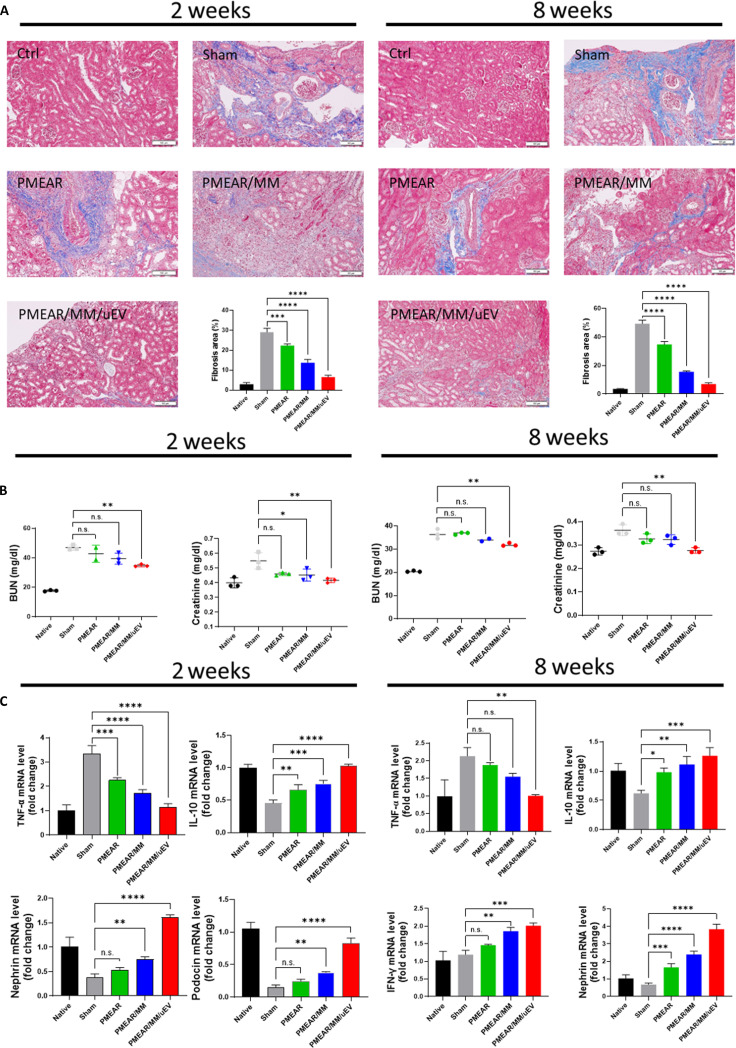
In vivo anti-fibrotic and anti-inflammatory effects of scaffolds. (A) Schematic and representative images of the 5/6 nephrectomy model Masson’s Trichrome staining at 2 and 8 weeks. Scale bars = 100 μm (*n* = 3). (B) Serum BUN and creatinine levels measured at 2 and 8 weeks postimplantation (*n* = 3). (C) Quantification of TNF-α, IL-10, nephrin, and podocin mRNA levels in kidney tissue (*n* = 3).

MT staining at 2 and 8 weeks revealed extensive collagen deposition in injury-only kidneys, confirming progressive fibrosis (Fig. 5A). Scaffold-treated groups showed markedly reduced fibrotic remodeling, with PMEAR/MM/uEV exhibiting the most significant suppression of collagen accumulation. This aligns with transcriptomic data showing down-regulation of pro-fibrotic genes and enrichment of ECM-remodeling pathways.

Serum blood urea nitrogen (BUN) and creatinine were measured (Fig. 5B). In the sham group, both markers were significantly elevated at 2 and 8 weeks, consistent with impaired kidney function. PMEAR/MM/uEV treatment reduced BUN and creatinine to near-control levels, indicating substantial functional recovery, while PMEAR/MM showed moderate improvement and PMEAR alone had the least effect.

We next assessed the immune response by quantifying mRNA levels of pro-inflammatory cytokine TNF-α and anti-inflammatory cytokine IL-10. TNF-α expression was significantly up-regulated in the sham group but reduced in all scaffold-treated groups, particularly those containing MM cells and uEVs. Conversely, IL-10 expression was elevated in the scaffold-implanted groups, reflecting enhanced anti-inflammatory activity (Fig. 5C). These cytokine profiles suggest that the scaffold system modulates the immune microenvironment to favor tissue recovery.

To further investigate nephron preservation, we measured mRNA expression of nephrin and podocin, essential markers of podocyte integrity and glomerular filtration barrier function [46]. Both genes exhibited progressive up-regulation in scaffold-treated animals, supporting the notion that the bioactive scaffolds aid in the maintenance or restoration of renal architecture.

Taken together, these results demonstrate that the PMEAR/MM/uEV scaffold exerts sustained antifibrotic and anti-inflammatory effects in vivo, as evidenced by reduced collagen deposition, improved renal function biomarkers, and a cytokine profile characterized by increased IL-10 and decreased TNF-α expression. Overall, PMEAR/MM/uEV demonstrated the most robust and sustained anti-fibrotic, anti-inflammatory, and nephron-protective effects, consistent with the multipathway regenerative activation identified in RNA-seq and GO/KEGG analyses.

### Immunohistochemical and histological evidence of renal recovery induced by biofunctional scaffolds

To assess cellular-level recovery of renal tissue following scaffold implantation, we performed immunofluorescence staining for key structural and regenerative markers, as well as histological evaluation of glomerular restoration.

**Fig. 6.**
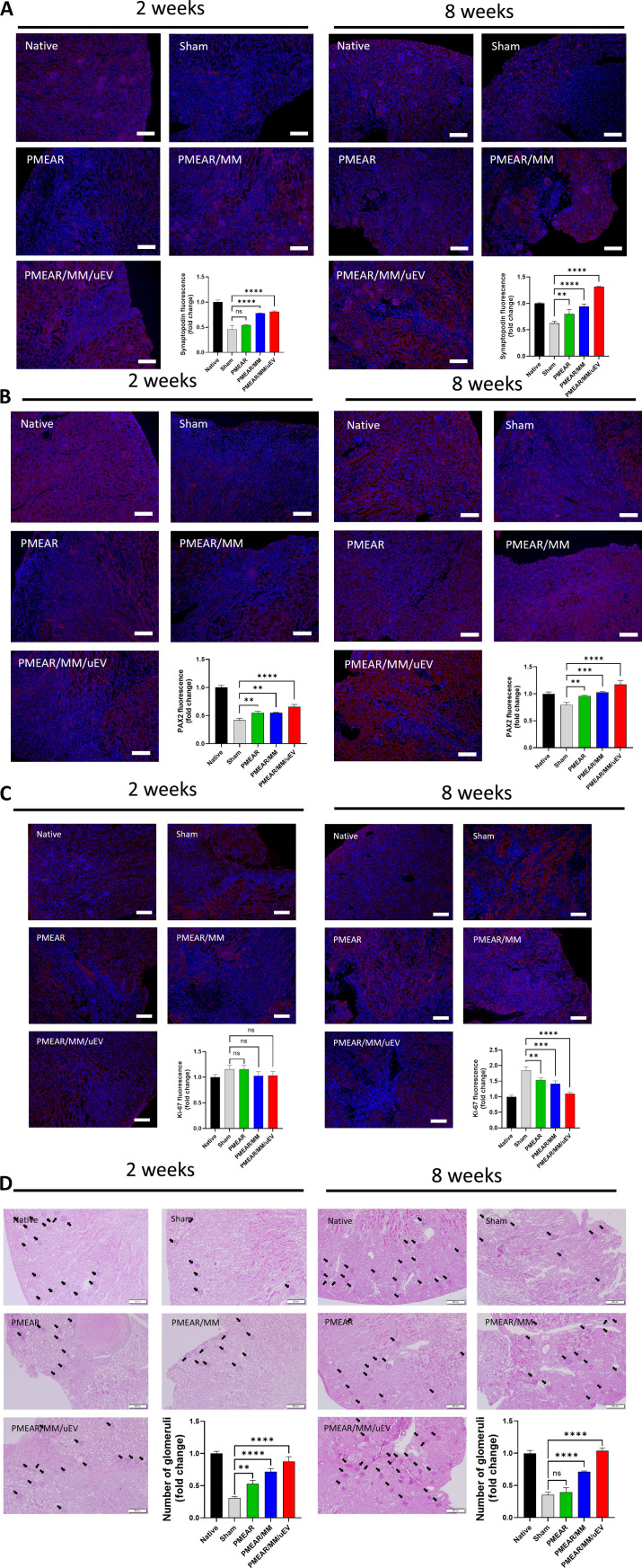
In vivo regenerative effects of EV-loaded scaffolds. (A) Synaptopodin immunofluorescence in kidney sections at 2 and 8 weeks postimplantation. Scale bars = 100 μm (*n* = 3). (B) Pax2 expression in renal tissue. Scale bars = 100 μm (*n* = 3). (C) Ki-67 staining showing proliferative activity. Scale bars = 100 μm (*n* = 3). (D) H&E-stained glomeruli and corresponding quantification across groups. Scale bars = 200 μm (*n* = 3).

Synaptopodin, an actin-associated protein expressed exclusively in podocyte foot processes, plays a central role in maintaining podocyte cytoskeletal integrity and glomerular function [28]. Fluorescence intensity of synaptopodin was markedly decreased in the injury-only group at both 2 and 8 weeks, indicating significant podocyte loss. In contrast, scaffolds loaded with MM cells and uEVs (PMEAR/MM/uEV) induced a strong increase in synaptopodin expression, maintaining a fluorescence level comparable to the native control at both time points (Fig. 6A). This suggests that the PMEAR/MM/uEV scaffold supports the preservation and possible regeneration of podocyte structures.

Pax2, a transcription factor essential for nephron development and re-expressed during renal repair processes, also showed elevated expression in the PMEAR/MM/uEV group, mirroring the trend observed with synaptopodin (Fig. 6B) [32]. Pax2 up-regulation in this context may reflect scaffold-driven activation of nephron progenitor-like cells and repair-associated signaling pathways [47].

Cellular proliferation was examined by immunostaining for Ki-67, a nuclear protein expressed during active phases of the cell cycle [33]. At 2 weeks, Ki-67 expression showed no significant differences across groups. However, at 8 weeks, the sham group exhibited a sharp increase in Ki-67+ nuclei, potentially indicating ongoing compensatory proliferation due to unresolved injury. In contrast, scaffold-implanted groups, particularly those with MM and uEV components, showed reduced Ki-67 expression, implying that early structural recovery and inflammation suppression may have alleviated the need for prolonged proliferative responses (Fig. 6C).

To evaluate structural preservation at the organ level, we counted the number of visible glomeruli in H&E-stained sections. Glomerular density was significantly higher in the PMEAR/MM/uEV group than in the injury control group, indicating better nephron preservation and reduced fibrotic remodeling (Fig. 6D). This result is consistent with previous studies showing that fibrosis and glomerular dropout are hallmarks of progressive renal failure [48].

Taken together, these findings support that the EV- and cell-loaded hybrid scaffold promotes tissue-level regeneration in damaged kidneys, as demonstrated by restored podocyte markers, enhanced regenerative signaling, reduced injury-induced proliferation, and preserved nephron structures.

## Conclusion

This study demonstrates that the biofunctional hybrid scaffold (PMEAR/MM/uEV), integrating a porous PLGA-based matrix, porcine-derived ECM, MH-RA, MM-like cells, uEV, and bioactive molecules (resveratrol and adapalene), offers a multifaceted platform for CKD repair. Transcriptomic profiling and functional enrichment analyses revealed that this scaffold uniquely activates molecular programs associated with angiogenesis, ECM stabilization, immune modulation, oxidative stress defense, and cytokine-mediated repair.

In vitro assays confirmed these predictions by showing that PMEAR/MM/uEV scaffolds enhanced wound closure, up-regulated nephron-specific podocyte markers (synaptopodin, nephrin, and podocin), reduced intracellular ROS in oxidative stress conditions, and modulated cytokine profiles toward an anti-inflammatory phenotype by increasing IL-4 and reducing TNF-α, IL-6, and IL-8.

In vivo implantation in a 5/6 nephrectomy mouse model further validated these effects. PMEAR/MM/uEV-treated mice exhibited significantly improved renal function biomarkers (BUN and creatinine), reduced interstitial fibrosis, preserved glomerular architecture, and increased expression of podocyte markers alongside the developmental repair marker Pax2. These histological and molecular improvements were sustained over both early and late injury phases, accompanied by enhanced microvascular density, aligning with the scaffold’s angiogenic transcriptomic signature.

Collectively, these findings establish PMEAR/MM/uEV as the most effective configuration among tested scaffolds for kidney tissue regeneration, offering a synergistic combination of structural support, biochemical cues, immunomodulation, and antioxidant protection. This multifunctional approach holds strong translational potential for the development of next-generation regenerative therapies targeting CKD.

## Data Availability

The data that support the findings of this study are available from the corresponding author upon reasonable request.
